# Large and pristine films of reduced graphene oxide

**DOI:** 10.1038/srep18799

**Published:** 2015-12-22

**Authors:** Sung Il Ahn, Kukjoo Kim, Jura Jung, Kyung Cheol Choi

**Affiliations:** 1 Department of Engineering in Energy and Applied Chemistry, Silla University, Busan 617-736 (Republic of Korea); 2 Department of Electrical Engineering, KAIST, Daejeon 305-701 (Republic of Korea)

## Abstract

A new self-assembly concept is introduced to form large and pristine films (15 cm in diameter) of reduced graphene oxide (RGO). The resulting film has different degrees of polarity on its two different sides due to the characteristic nature of the self-assembly process. The RGO film can be easily transferred from a glass substrate onto water and a polymer substrate after injection of water molecules between the RGO film and glass substrate using an electric steamer. The RGO film can also be easily patterned into various shapes with a resolution of around ±10 μm by a simple taping method, which is suitable for mass production of printed electronics at low cost.

Highly oxygenated graphene, graphene oxide (GO), has been extensively researched as it can be functionalized with various molecules^1^,^2^,^3^, fabricated in composites with polymers^4^,^5^, and as large area films^6^,^7^,^8^. It can also be applied as paper-like materials with superior mechanical properties^9^,^10^, and offer the possibility of mass production. GO is known as a non-conductor due to the disconnected network of its conjugated carbon domains by oxygenated groups including alcohols, epoxies, and carboxylic groups. A reduced form of GO (RGO) with reasonable conductivity has been developed and characterized for electronic applications. One of the most interesting challenges in the research field of graphene related materials is to develop macroscopic scale films due to their potential applications in many areas. These include: graphene oxide paper for protective layers^11^, chemical filters^12^,^13^, and electrode of energy devices^9^, single or multi-layers films of graphene made by chemical vapor deposition or Langmuir–Blodgett assembly for stretchable and/or transparent electrodes^10^,^14^, and RGO papers for flexible and ultrathin electrodes^7^,^8^. We previously researched methods to obtain stable RGO or to control the oxygen content of GO in a solution without agglomeration of the RGO sheets and developed a hydrazine-based method to carefully control the degree of reduction of GO^15^. During the heat treatment of spin-coated GO to dry and reduce it further, a phenomenon of self-assembly of RGO sheets was observed on a certain area of the substrate. Here, we report a new self-assembly process of RGO and fabrication of wafer-sized RGO films using the method. We named the process a reaction-based self-assembly (RSA) process because GO with hydrazine and/or some of the un-reacted hydarzine were involved in the process.

## Results and Discussion

**Figure 1:**
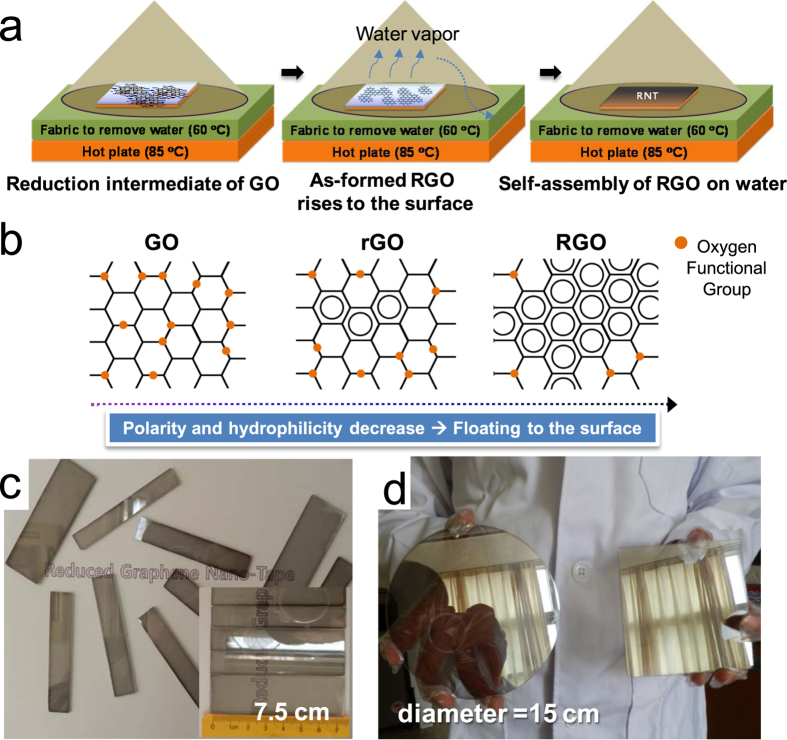
Schematic, structure, and various shapes of RGO films. (**a**) A schematic of reaction-based self-assembly of RGO, (**b**) A schematic of the structure of floating RGO on aqueous dispersion of GO with hydrazine during the RSA, (**c**) Bar-type RNTs with various widths on glass with a length of 7.5 cm, (**d**) Wafer sized RNTs on glass (15 cm in diameter; Supplementary Fig. S2 online shows various sized circle-type RNTs compared to a 5.25 inch Si wafer).

**Figure 2:**
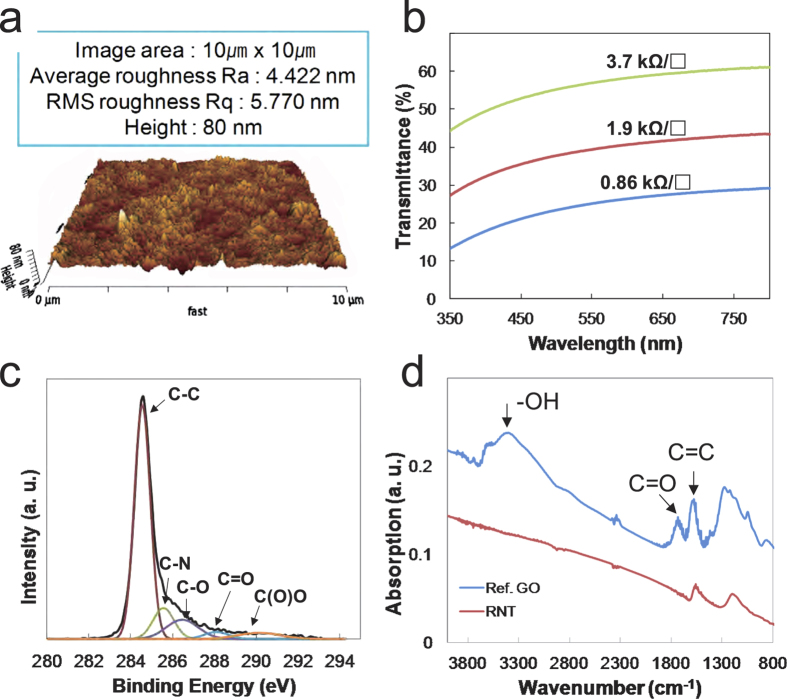
Physical and chemical characterization of RNT. (**a**) AFM image of the surface of the RNT after heat treatment at 200 ^o^C for 30 min, (**b**) Sheet resistances of RNTs with various transmittances after heat treatment at 200 ^o^C for 30 min, (**c**) XPS spectrum of the RNT fitted after a Shirley background correction, (**d**) FT-IR absorption spectra.

**Figure 3:**
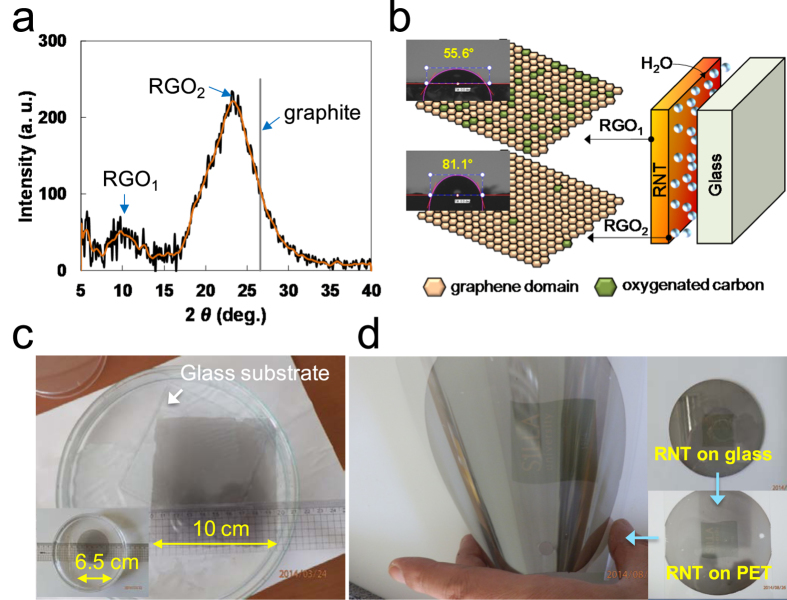
Polarity and transfer of RNT. (**a**) XRD spectrum of the RNT showing a major and minor peak at around 23.5 and 10 degree, respectively, (**b**) An expected structure of the as-formed RNT on a glass substrate (Photographic images are the contact angles of both sides), (**c**) Steaming off 10 cm square-type and 6.5 cm circle-type RNTs (the inset), (**d**) Transferring the RNT from glass onto a commercial PET film. Inset photos show the RNT on glass and the as-transferred RNT on PET (see Supplementary video1 online. Permission is granted to use the logo of Silla University.).

The RSA process differs from other self-assembly phenomena of graphene related materials in that the process dose not use a completely reduced form of GO and can occur at relatively high temperature (above 60 °C). It appears that the moderately slow reaction rate of hydrazine with GO allows the RSA process to take place (see Supplementary Fig. S1 online; photoluminescence spectra of GO dependent on reaction time)^15^,^16^. As shown in Fig. 1a, the RSA process converts reduction intermediates of GO to RGO as a first step, and RGO rises to the surface of the reaction mixture due to the decreased polarity and hydrophilicity of the RGO sheet described in Fig. 1b. It has been reported that graphene has 46.7 mJ/m^2^ of surface free energy, which is much smaller than GO (62.1 mJ/m^2^) and even graphite (54.8 mJ/m^2^)^17^. The as-formed RGO sheets are then self-assembled on the surface of the reaction mixture before aggregation of the RGO sheets. Hereafter, we refer to the self-assembled RGO film as “reduced graphene oxide nano-tape (RNT)” because of its unique features, which are explained later. The RSA process was successfully developed and by controlling the concentration of GO, temperature, humidity, and even air flowing over the substrate, an RNT with a diameter of 15 cm was obtained (Fig. 1c,d; Supplementary Fig. S2 online shows various sized circle-type RNT compared to a 5.25 inch Si wafer). The normal thickness and roughness of the RNT were around 80 and 5 nm, respectively, measured using atomic force microscopy (AFM) as shown in Fig. 2a. According to previous reports, the sheet resistances of RGO films vary from tens of kΩ/□ to a few kΩ/□ depending on the reduction methods, GO structures and coating methods^18^,^19^,^20^,^21^. As-prepared RNT shows about 10 kΩ/□ at 37% transmittance (at 550 nm). Heat treatment of the film at 200 °C for 30 min decreased the sheet resistance to 0.86 kΩ/□ at 25% transmittance for the film with a thickness of 80 nm as shown in Fig. 2b. XPS spectrum in Fig. 2c indicates that as-formed RNT has various functional group in RGO sheets; C-N at 285.6 eV, C-O at 286.5 eV, and C=O at 280.1 eV. The C : O ratio of the as-formed RNT was 8.7 : 1 based on the spectrum. The FT-IR spectra in Fig. 2d shows two strong absorptions at 1570 cm^−1^ assigned to aromatic C=C vibration and around 1200 cm^−1^ assigned to several possible functional groups such as C-O vibration (ether or ester), C-N, and S-O, while -OH, C=O, and COOH groups disappeared during the RSA process. The XRD spectrum of RGO in Fig. 3a shows two broad peaks at around 10° (RGO_1_) and 23° (RGO_2_). As described in Fig. 3b, less reduced sheets of GO appear to be preferentially located on the upper side of the RNT (RGO_1_) due to having less chances to contact with hydrazine in the reaction mixture. Compared to the upper face of the RNT, the RGO sheets placed on the underside appears to have less oxygenated sites. The different contact angle of each side clearly shows the different polarity on both sides (see inset in Fig. 3b).

**Figure 4:**
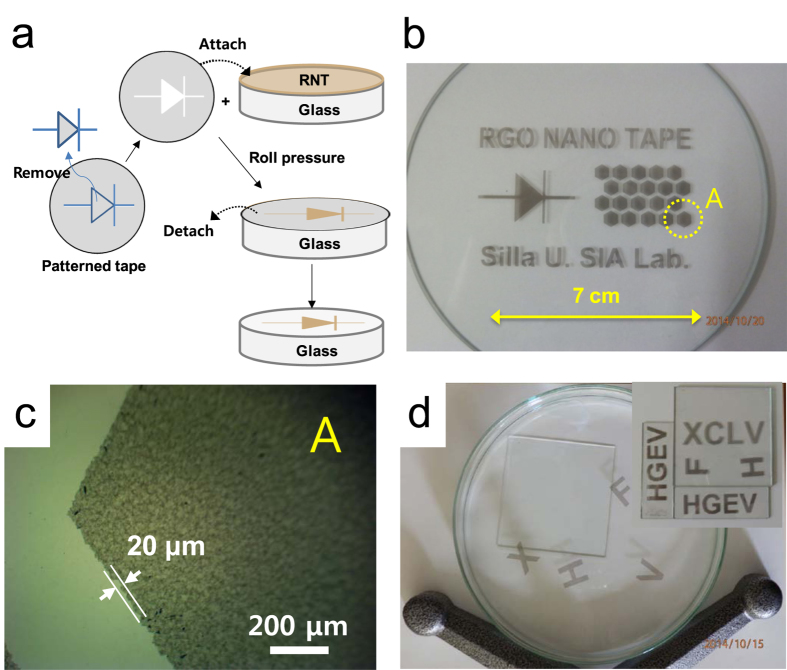
Patterning and transferring of RNT. (**a**) Process flow for the patterning of the RNT, (**b**) Patterned RNT using the process described in a, (**c**) A microscope image of the edge of the hexagonal shape RNT showing the roughness of the pattern (area A in b), (**d**) Patterned RNT transferred from the glass substrate onto water (the inset is a photograph of patterned RNT with various characters used in this transfer).

Although we successfully obtained an extremely large and pristine RNT using the RSA process, detaching it for further applications without damage requires a new approach, due to the adhesion force between the RNT and the glass substrate. Not surprisingly, the separation of films of graphene related materials from substrates is one of the key technologies under development in this research field. Reported approaches include an etching technique for peeling a CVD graphene film from a nickel substrate^6^ and methods to peel an RGO film from a copper substrate^22^,^23^. Free-standing papers have also been reported for GO and RGO films with a thickness over 1 μm^4^,^24^. Instead of using an etching or dissolving technique, which can cause potential contamination of the transferred films, the detaching process used in this study simply exploits the tendency of the RNT film to exist at the surface of water, which is based on two characteristics: RGO sheet has a lower polarity than water, and a certain amount of water molecules exist between the RNT and the glass substrate dependent on a drying condition. When RSA was initially attempted, the process was carried out in an open space on a hot plate. The resulting film had wave patterns and did not peel off well, owing to the strong adhesion force between the RNT and the glass substrate. By isolating the gas space above the substrate with a conical shaped glass cover as described in Fig. 1a, the process resulted in a pristine RNT. It appears that the cover minimized the influence of introducing air flow, which could disturb the equilibrium of self-assembly of the RGO sheets on the surface of the reaction mixture. Fortunately, in the case of the small sized RNT, we found that the saturated humidity sustained by the cover diminished the adhesive strength of the as-formed RNT, which allowed it to be easily peeled off on water. On the other hand, larger RNT samples had a larger adhesion force to the glass substrate, resulting in broken pieces of RNT when it was transferred onto water. To solve this problem, we steamed the as-formed RNT to insert water molecules between the RNT and glass substrate. Using the steamed RNT, we peeled off 10 cm square and 6.5 cm circular RNTs on water without cracks as shown in Fig. 3c. We found that it was possible to move the RNT toward a specific direction by rolling a glass tube on the distilled water. Using this phenomenon, the RNT could be transferred onto a glass substrate from water (see Supplementary Fig. S3 online to visualize the process of transferring the RNT). Research to develop inexpensive and scalable patterning methods for graphene related materials has been one of the hottest topics in this field. Many patterning methods have been considered to date, including lithography using a patterned mask^25^,^26^, transfer printing^27^,^28^, direct chemical vapor deposition (CVD) growth of patterned graphene on a patterned catalyst^10^,^29^, and barrier guided CVD growth^30^,^31^. However, scalability and production hurdles must be overcome for wider application of these patterning methods. We have made RNTs in various sized square shapes by a simple physical method using a cutter (see Supplementary Fig. S4 online), and transferred the entire RNT film from a glass substrate to a commercial PET film for the protection of tablets or smart phones as shown in Fig. 3d (see Supplementary video1 online). Based on these properties, we introduce a simple taping method to pattern the RNT. Following the process flow of the method illustrated in Fig. 4a, large scale RNT patterns including characters, lines, and hexagon shapes have been demonstrated, as shown in Fig. 4b. In the image, the patterns are slightly difficult to see due to the shadows of the patterns underneath the glass substrate, but closer examining reveals well defined lines with around ±10 μm roughness based on the microscope image of the hexagonal edge (see Fig. 4c). In addition, the patterns could be transferred onto water, as shown in Fig. 4d and then transferred to other substrates, or directly transferred to various substrates using a similar taping method. Although this method should be improved to obtain patterns with a micro-scale, it has several advantages in that it is scalable, low cost, and fast. Most importantly, we believe that it can be applied to a continuous process system.

## Methods

### Preparation of GO

First, 4 g of graphite (Aldrich, <20 μm) was dispersed into 150 ml of concentrated H_2_SO_4_ (Dae Jung, 96%) dissolved with 2 g of NaNO_3_ (Aldrich, 99%) at room temperature and stirred using a magnetic stirrer. Second, 12 g of KMnO_4_ (Aldrich, 99%) was added carefully to the mixture in an ice bath. Then, the temperature of the stirrer was adjusted to 35 °C and maintained for 1 h. The reaction was terminated by adding 300 ml of distilled water slowly for 30 min and then H_2_O_2_ (Dae Jung, 30 wt %) until bubbles in the mixtures disappeared, which indicated the disappearance of un-reacted KMnO_4_. The oxidized graphite was centrifuged and cleaned repeatedly with distilled water (more than 20 times), until the centrifuged solution didn’t show precipitations with BaCl_2_ solution. Then the residues were dispersed into 150 ml of distilled water in a beaker and sonicated for 10 min using a sonicator (20 kHz and 200 W). After that the mixture was centrifuged and the supernatant containing the exfoliated GO was transferred to vials.

### RSA process

Using an ice bath, we cooled a 30 ml mixture containing 0.012 wt% GO in a beaker and then slowly dropped 6 ml of 8 wt% aqueous diluted solution of hydrazine (80 wt%, Daejung). The mixture was stirred for 15 min and then kept in an ice bath. On a hot-plate with ±1 °C accuracy at 200 °C, we placed 6 layers of a clean wiper to slowly vent water vapor during the RSA process of RGO and then, adjusted the horizontality of the fabric surface using a level with 0.02 mm accuracy. The process temperature was around 60 °C measured at the surface of the venting fabric when the temperature of the hot plate was set to 85 °C. After the temperature of the fabric reached to 60 °C, we placed a glass substrate on it, waited until the temperature of the substrate reached around 40 °C, and then dropped the mixture of partially reduced GO onto glass substrate until it covered the entire surface. The amount of the reaction mixture was about 0.055 ± 0.002 g/cm^2^. Finally, the process environment was isolated by a glass cover of a conical shape to protect the reaction area from unexpected air flow, and water drops, which was formed by condensation along its inside wall. The self-assembly process was terminated after about 80 min or after observing the disappearance of a circular mark of water (see Supplementary Fig. S5). The thickness of the RGO film was controlled by GO content within a ±10% change in the reaction mixture. More than ±10% change in GO content resulted in an imperfect RGO films or aggregations of RGO sheets.

### Patterning of RNT

The as-formed RNT on a glass substrate was exposed to water vapor at a temperature below 60 °C using a commercialized electric steamer for 3 h to inject water molecules between the RNT film and the glass substrate. Then, it was dried at a temperature below 40 °C for 5 h to remove moisture on the surface of the sample. Engraved patterns on a commercial waterproof label paper were prepared using an electronic cutting tool. Then, we attached the patterned paper to the steamed RNT, pressured it using a roll to remove air bubbles between the tape and RNT, and then detached the patterned tape as described in Fig. 4a.

### Characterizations

The transmittance and sheet resistance of the RNT film were measured using a UV-Vis spectrometer (K-MAC, 2100V) and the four point probe method, respectively. The RNT was further characterized using Fourier transform infrared spectroscopy (FT-IR; Bruker, IFS66v/S), X-ray photoelectron spectroscopy (XPS; VG Scientific), X-ray diffraction (XRD; Rigaku, D/MAX 2500), and atomic force microscope (AFM; Nanowizard, SFM). For the measurement of XPS and XRD, the RNT film was transferred from a glass substrate to a silicon wafer and, for FT-IR, to ZnSe crystal.

## Additional Information

**How to cite this article**: Ahn, S. *et al.* Large and pristine films of reduced graphene oxide. *Sci. Rep.* **5**, 18799; doi: 10.1038/srep18799 (2015).

## Supplementary Material

Supplementary Information

Supplementary video
