# Supplementary material for: Large and pristine films of reduced graphene oxide
Source: Sci Rep. 2015 Dec 22;5:18799. doi: 10.1038/srep18799 (PMC4686933; doi:10.1038/srep18799)
Supplement: Supplementary Information [file srep18799-s1.doc]

**Supplementary Information**

**Large and pristine films of reduced graphene oxide**

Sung Il Ahn,^1,*^ Kukjoo Kim,^2^ Jura Jung,^1^ and Kyung Cheol Choi^2^

^1^ Department of Engineering in Energy and Applied Chemistry, Silla University, Busan 617-736 (Republic of Korea)

^2^ Department of Electrical Engineering, KAIST, Daejeon 305-701 (Republic of Korea)

*^*^* Corresponding authors. E-mail: [siahn@silla.ac.kr](mailto:siahn@silla.ac.kr)

**PL spectra**; When the GO was reacted with a reducing agent, the PL intensity decreased due to the increase of the size of localized clusters of sp^2^ carbon on the RGO. Observing this phenomenon, we can easily detect the progress of the reduction of GO with hydrazine in the reaction mixture. The PL spectra of the sample excited at 463 nm in Fig. S1 show a clear trend of intensity reduction as the reaction time increases. In addition, the degree of decrease of the PL intensity indicates that the reduction of GO continues for a long period of over five days.


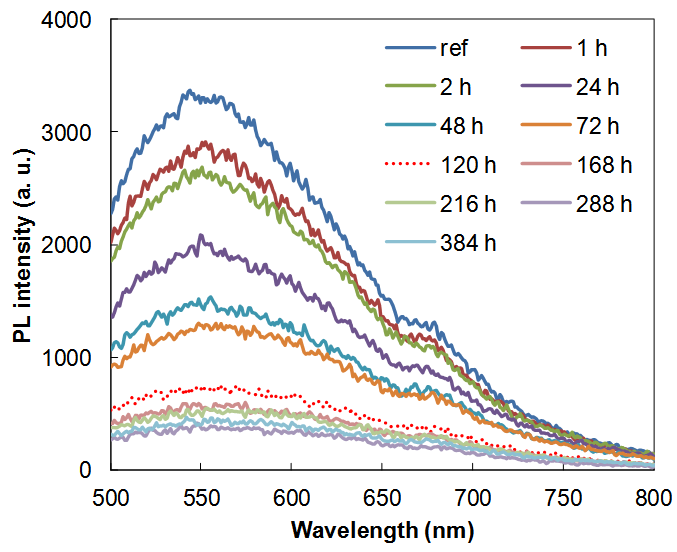


Figure S1. PL spectra of GO according to the reaction time measured *in situ*, excited at 463 nm. The decrease of the PL intensity indicates that the reduction of GO with hydrazine continues for more than five days. For this experiment, we cooled 30 ml solution with 0.02 wt% GO in a beaker and then added 0.2 ml of 0.8 wt% hydrazine.


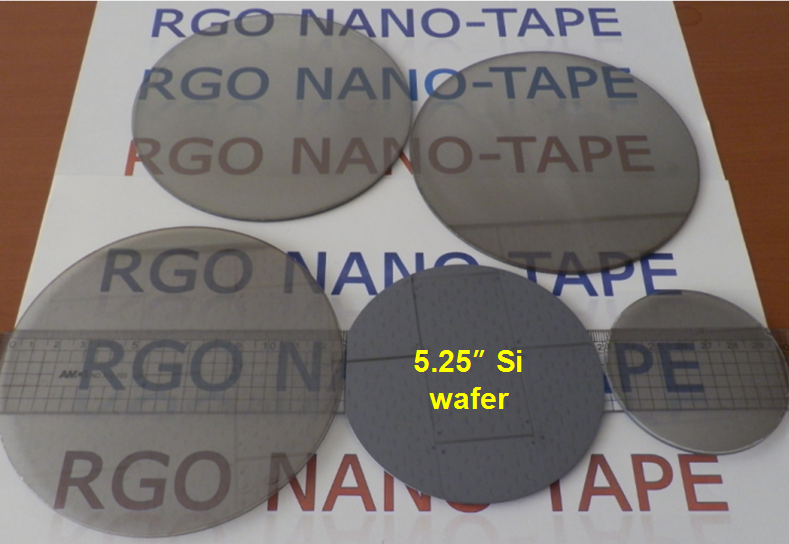


Figure S2. Various sized circular RNTs compared to a 5.25 inch Si wafer

Figure S3. Transfer of RNT onto a glass substrate from water.

Figure S4. Photographs of peeled RNT cut into 5 pieces excluding edge cuts by a simple cutter

Figure S5. Photographs of the dry process after self-assembly of RGO on the reaction mixture; a. Initial stage of the RSA process, b. dry process after self-assembly of RGO showing a circular mark of water.

**Video Legend**

Video 1: Transferring the RNT from glass onto a commercial PET film.
